# Supplementary material for: Development of an optimized, non‐stem cell line for intranasal delivery of therapeutic cargo to the central nervous system
Source: Mol Oncol. 2023 Dec 26;18(3):528–46. doi: 10.1002/1878-0261.13569 (PMC10920084; doi:10.1002/1878-0261.13569)
Supplement: Supplementary file 1 — Fig. S1. Cell line authentication data. Fig. S2. Cell motility of PAR and FR cells determined by live cell imaging. Fig. S3. Proliferation of PAR, FR, and FR/TK cells. Fig. S4. GCV vulnerability of FR and FR/TK cells. Fig. S5. Uncropped immunoblots as shown in partial in Fig. 5. Fig. S6. Representative microphotographs of migrated LX2 cells from olfactory epithelium (OE) to the olfactory bulb (OB) of the mice. Fig. S7. In vivo in brain migration of shuttle cells. Table S1. Abbreviations, names and function of genes presented in Fig. 7C. [file MOL2-18-528-s001.zip › mol213569-sup-0001-FigureS1.pdf]

Eurofins Genomics Europe Applied Genomics GmbH, Anzinger Str. 7 a, D-85560 Ebersberg

Prof. Dr. Ulrike Naumann
University Tübingen, Hertie Institute
Otfried-Müller-Straße 27
72076 Tübingen
Germany

eurofins | Genomics

SOP_APG_Zelllinienauthentizität_A04_2.0

Person in charge: Dr. Torsten Brendel
Report date: 25.05.2023
Sample received on: 17.05.2023
Start / End of Analysis: 17.05.2023/25.05.2023

Analytical Report:
Cell Line Authentication Test
Order ID: 11108431290

Method:

Genetic characteristics were determined by PCR-single-locus-technology.
16 independent PCR-systems D8S1179, D21S11, D7S820, CSF1PO, D3S1358, TH01, D13S317, D16S539, D2S1338, AMEL, D5S818, FGA, D19S433, vWA, TPOX and D18S51 were investigated.
(ASN-0002 core markers are colored grey, Thermo Fisher, AmpFlSTR® Identifiler® Plus PCR Amplification Kit)
In parallel, positive and negative controls were carried out yielding correct results.
Method details are given in **SOP_APG_Zelllinienauthentizität_2.0**

Result:

	**LN-229**	**U87MG**		**R49 GSCs**		**LX-2**	**HB1.F3**	**HEK293**	**HEK293FT**
**Client Sample Name**	LN-229	U87	[redacted]	R49 GSC pass44 v. 2018	[redacted]	LX-1 pass 6	HB1.F3 pass 33 v. 2014	293 MX	293 FT
**Sample Code**	CL00013070	CL00013071	[redacted]	CL00013068	[redacted]	CL00013073	CL00013074	CL00013075	CL00013076
D8S1179	13,13	10,11	[redacted]	11,11	[redacted]	13,13	14,14	12,14	12,14
D21S11	29,30	28,32.2	[redacted]	29,30	[redacted]	28,31	28,28	28,30.2	30.2,30.2
D7S820	8,11	8,9	[redacted]	12,12	[redacted]	11,11	8,11	11,12	11,11
CSF1PO	12,12	10,11	[redacted]	9,10	[redacted]	10,12	11,11	11,12	7,12
D3S1358	16,17	16,17	[redacted]	14,16	[redacted]	13,15	15,15	15,17	15,17
TH01	9.3,9.3	9.3,9.3	[redacted]	8,9	[redacted]	9.3,9.3	6,9	7,9.3	7,9.3
D13S317	10,11	11,11	[redacted]	11,11	[redacted]	11,13	8,9	12,12	12,14
D16S539	12,12	12,12	[redacted]	11,13	[redacted]	13,13	9,12	9,13	9,13
D2S1338	19,20	20,23	[redacted]	24,24	[redacted]	17,17	19,22	19,19	19,19
D19S433	12,17.2	15,15.2	[redacted]	13,13	[redacted]	13,15.2	14,14.2	15,18	15,18
vWA	16,19,20	15,17	[redacted]	18,18	[redacted]	17,17	16,19	16,19	16,19
TPOX	8,8	8,8	[redacted]	8,12	[redacted]	8,9	10,11	11,11	11,11
D18S51	13,15	13,13	[redacted]	18,18	[redacted]	12,12	16,19	17,17	17,18
AMEL	X,X	X,X	[redacted]	X,X	[redacted]	X,Y	X,X	X,X	X,X
D5S818	11,12	11,12	[redacted]	11,11	[redacted]	11,12	11,12	8,9	8,9
FGA	23,23	18,24	[redacted]	25,25	[redacted]	21,26	23,24	23,23	23,23
			[redacted]		[redacted]				
**Cellosaurus ID:**	**CVCL_0393**	**CVCL_0022**		**not available**		**CVCL_5792**	**CVCL_LJ44**	**CVCL_0045**	**CVCL_6911**

Supplementary Figure 1: Cell line authentication
