# Supplementary material for: Development of an optimized, non‐stem cell line for intranasal delivery of therapeutic cargo to the central nervous system
Source: Mol Oncol. 2023 Dec 26;18(3):528–46. doi: 10.1002/1878-0261.13569 (PMC10920084; doi:10.1002/1878-0261.13569)
Supplement: Supplementary file 1 — Fig. S1. Cell line authentication data. Fig. S2. Cell motility of PAR and FR cells determined by live cell imaging. Fig. S3. Proliferation of PAR, FR, and FR/TK cells. Fig. S4. GCV vulnerability of FR and FR/TK cells. Fig. S5. Uncropped immunoblots as shown in partial in Fig. 5. Fig. S6. Representative microphotographs of migrated LX2 cells from olfactory epithelium (OE) to the olfactory bulb (OB) of the mice. Fig. S7. In vivo in brain migration of shuttle cells. Table S1. Abbreviations, names and function of genes presented in Fig. 7C. [file MOL2-18-528-s001.zip › mol213569-sup-0006-FigureS6.pdf]

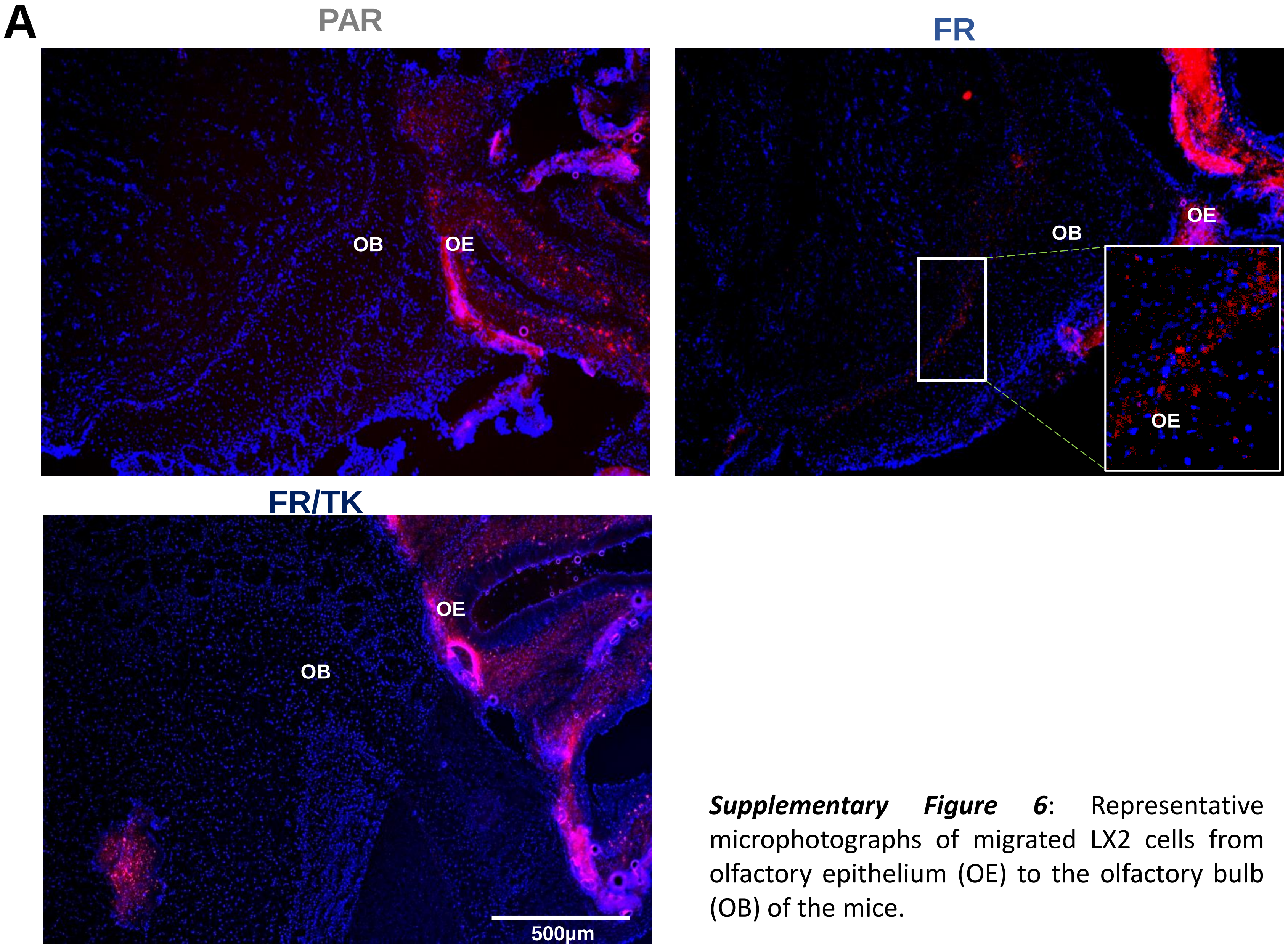

Supplementary Figure 6: Representative microphotographs of migrated LX2 cells from olfactory epithelium (OE) to the olfactory bulb (OB) of the mice.
