# Supplementary material for: Development of an optimized, non‐stem cell line for intranasal delivery of therapeutic cargo to the central nervous system
Source: Mol Oncol. 2023 Dec 26;18(3):528–46. doi: 10.1002/1878-0261.13569 (PMC10920084; doi:10.1002/1878-0261.13569)
Supplement: Supplementary file 1 — Fig. S1. Cell line authentication data. Fig. S2. Cell motility of PAR and FR cells determined by live cell imaging. Fig. S3. Proliferation of PAR, FR, and FR/TK cells. Fig. S4. GCV vulnerability of FR and FR/TK cells. Fig. S5. Uncropped immunoblots as shown in partial in Fig. 5. Fig. S6. Representative microphotographs of migrated LX2 cells from olfactory epithelium (OE) to the olfactory bulb (OB) of the mice. Fig. S7. In vivo in brain migration of shuttle cells. Table S1. Abbreviations, names and function of genes presented in Fig. 7C. [file MOL2-18-528-s001.zip › mol213569-sup-0008-TableS1.pdf]

Supplementary Table

Cell Adhesion		Focal Adhesion	
**Gene**	**Name and Function**	**Gene**	**Name and Function**
ADGRE5	Adhesion G protein-coupled receptors, mediate cell-cell interactions.	ITGBL1	Integrin subunit beta like 1, integrin-related protein that is a member of the EGF-like protein family
ICAM1	Intercellular Adhesion Molecule 1, intracellular adhesion molecule	SLC4A2	solute carrier family 4 member 2, membrane transport protein
PCDHGB4	Protocadherin Gamma Subfamily B, 4, involved in cell adhesion	HYOU1	Hypoxia upregulated-1, facilitates invasion
NID2	Nodogen-2, basal laminal protein	ITGB1	Integrin beta 1, when associated with alpha-7 integrin, regulates cell adhesion and laminin matrix deposition
CDH13	Cadherin-13, responsible for selective cell recognition and adhesion	ADAM17	ADAM Metallopeptidase Domain 17, membrane-anchored metalloproteinase
PCDHGC3	Protocadherin Gamma Subfamily C3, establishment and function of specific cell-cell connections in the brain	FLNA	Flamin-A, regulates reorganization of the actin cytoskeleton
JCAD	Junctional protein assoxiated with coronary artery disease, cell to cell junction protein	CALR	Calreticulin, involved in cell adhesion
EMB	Embigin, transmembrane glycoprotein	ITGAV	Integrin Subunit Alpha V, transmembrane receptor involved cell adhesion
HPSE	Heparanase, acts within the extracellular matrix to degrade polymeric heparan sulfate	CD9	Tetraspanin-29, shown to increase migration
CD33	Siglec-3, sialoadhesin molecule	RHGAP24	Rho-GTPase activating protein 24, negative regulator of the Rho signaling
ADAM22	ADAM Metallopeptidase Domain 22, membrane-anchored protein	TEK	Tyrosine-protein kinase, acts as cell-surface receptor
ITGA11	Integrin Subunit Alpha 11, involved in the differentiation of stellate cells	ITGA11	Integrin Subunit Alpha 11, involved in the differentiation of stellate cells

Cell Junction		Negative Regulation of Cell Population Proliferation	
**Gene**	**Name and Function**	**Gene**	**Name and Function**
DLG3	Discs Large MAGUK Scaffold Protein 3, member of the membrane-associated guanylate kinase (MAGUK) superfamily	RBM38	RNA Binding Motif Protein 38, tumor-suppressor by stabilizing the p53-mdm2 loop function
CDK5RAP2	CDK5 Regulatory Subunit Associated Protein 2, Involved in	DDR1	Discoidin Domain Receptor Tyrosine Kinase 1, improtant for

	regulation of mitotic spindle orientation		communication of cells with their microenvironment
DMD	Dystrophin, stabilizes the plasma membrane	DLG3	Discs Large MAGUK Scaffold Protein 3, member of membrane-associated guanylatkinase-superfamily
YAP1	Yes-associated protein 1, transcriptional regulator involved also in the transcription of genes that regulate cell migration	FUZ	Fuzzy Planar Cell Polarity Protein, involved in directional cell movement
SPTBN1	Spectrin Beta, Non-Erythrocytic 1, actin crosslinking and molecular scaffold protein that links the plasma membrane to the actin cytoskeleton,	SMAD4	SMAD Family Member 4, involved in transmitting signals from the cell surface to the nucleus, especiall involved int TGF-β signaling
ESCO2	Establishment Of Sister Chromatid Cohesion N-Acetyltransferase 2, involved in the establishment of sister chromatid cohesion during S phase	RAPGEF2	Rap guanine nucleotide exchange factor 2, activates Rap and Ras family of small GTPases by exchanging bound GDP for free GTP
DCP2	Decapping MRNA 2, required for degradation of mRNAs	S100A11	S100 Calcium Binding Protein A11, accelerates the entry of β-Catenin into the nucleus
LCP1	Lymphocyte Cytosolic Protein 1, enables actin filament binding activity	TGFBP7	transforming growth factor β binding protein 7, also named LTBS, component of the extracellular matrix
JCAD	Junctional protein assoxiated with coronary artery disease, cell to cell junction protein	AR	Androgen Receptor, ligand-activated nuclear transcription factor
TES	Testin LIM Domain Protein, tumor suppressor	GATA3	zinc finger transcriptional factor GATA3 that is involved in the activation of HepSCs
CHRM2	Cholinergic Receptor Muscarinic 2, located in the postsynaptic cell membrane	CD33	Siglec-3, sialoadhesin molecule
TENM2	Teneurin Transmembrane Protein 2, promotes the formation of filopodia	SFRP2	Secreted Frizzled Related Protein 2, controls apoptosis, cell fate and Wnt pathway

**Collagen-containing Extracellular Matrix**		**Positive Regulation of Cell Migration**	
**Gene**	**Name and Function**	**Gene**	**Name and Function**
MFGE8	Lactadherin, enables integrin binding activity, strongly inhibits TGFβ signaling	PTK2B	cytoplasmic protein tyrosine kinase 2B, involved in calcium-induced regulation of ion channels
PSAP	Prosaposin, precursor of several small nonenzymatic glycoproteins termed 'sphingolipid activator proteins' that assist in lysosomal hydrolysis of sphingolipids	PIK3CD	Phosphatidylinositol 4,5-bisphosphate 3-kinase catalytic subunit delta isoform, plays a role in B-cell development, proliferation, migration

CDH13	Cadherin-13, responsible for selective cell recognition and adhesion	PDGFA	Platelet Derived Growth Factor Subunit A, common mediator of cell migration
THBS2	Thrombospondin 2, mediates cell-to-cell and cell-to-matrix interactions	SEMA3D	semaphorin 3D, involved in cell migration
NID2	Nodogen-2, basal laminal protein	SEMA6D	semaphorin 6D, involved in cell migration
HSPG2	Heparan Sulfate Proteoglycan 2/Perlecan, cross-links many extracellular matrix components and cell-surface molecules	PIK3R3	Phosphoinositide-3-Kinase Regulatory Subunit 3, art of regulatory domain of PI3K
PCOLCE	procollagen C-Endopeptidase Enhancer, binds and drives the enzymatic cleavage of type I procollagen	PLAU	Plasminogen Activator, Urokinase, associated with cell migration and invasion
COL11A2	Collagen Type XI Alpha 2 Chain, produces one component of this type of collagen, called the pro-alpha2(XI) chain. Type XI collagen adds structure and strength to tissues	PDGFRA	Platelet-derived growth factor receptor 2, PDGF-PDGFR network differentially regulates cell fate, migration, proliferation, and cell cycle progression
LUM	Lumican, extracellular matrix protein	SMO	Smoothened, can positively regulate proliferation and migration
CPA3	Carboxypeptidase A3, zinc metalloprotease	MDK	Midkine, exerts activities such as cell proliferation, migration and angiogenesis
ANGPT1	Angiopoietin 1, secreted glycoprotein that belongs to the angiopoietin family	MCAM	Melanoma Cell Adhesion Molecule,
		KIT	c-Kit/CD117, tyrosine kinase, plays a major role in melano-cytic migration
**Cytoskeleton**		**Regulation of Cell Population Proliferation**	
**Gene**	**Name and Function**	**Gene**	**Name and Function**
KIF21A	Kinesin Family Member 21A, member of the KIF4 subfamily of kinesin-like motor proteins	PTK2B	cytoplasmic protein tyrosine kinase 2B, involved in calcium-induced regulation of ion channels
LPXN	Leupaxin, member of the paxillin superfamily	TNFRSF9	TNF Receptor Superfamily Member 9, contributes to clonal expansion, survival, and development of T cells
DAPK1	Death-associated protein kinase 1, critical component in the ER stress-induced cell death pathway	PTCH1	Protein Patched Homolog 1, prevents cells from proliferating in an uncontrolled way
PCLO	Piccolo Presynaptic Cytomatrix Protein, part of the presynaptic cytoskeletal matrix	TES	Testin LIM Domain Protein, tumor suppressor

KIAA1217	Sickle Tail Protein Homolog, required for normal development of intervertebral disks
CCDC69	Coiled-Coil Domain Containing 69, regulator of central spindle formation during cytokinesis
ADD1	Adducin 1, cytoskeleton protein
HTRA2	HtrA serine peptidase 2, regulates autophagy
DMTN	Dematin Actin Binding Protein, actin binding and bundling protein
FSCN1	Fascin Actin-Bundling Protein 1, organizes F-actin into parallel bundles
RHOB	Ras homolog gene family, member B, Rho-GTP bindung protein, Enables GDP binding activity
BEX4	Brain Expressed X-Linked 4, functions as tumor suppressor

Extracellular Matrix Organization

Gene	Name and Function
COL4A2	Collagen Type IV Alpha 2 Chain, associated with laminin, entactin, and heparan sulfate proteoglycans
ABI3BP	ABI Family Member 3 Binding Protein, extracellular matrix protein
TGFBI	Transforming Growth Factor Beta Induced, plays a role in cell adhesion and migration
ADAMTSL4	ADAMTS Like 4, regulates cell migration and attachment
FOXF1	Forkhead Box F1, enables RNA polymerase II cis-regulatory region sequence-specific DNA binding activity
CRISPLD2	Cysteine Rich Secretory Protein LCCL Domain Containing 2, high affinity for lipopolysaccharide

ANXA1	Annexin A1, membrane-localized protein that binds phospholipids
CHST11	Carbohydrate Sulfotransferase 11, generateschondroitin-4-sulfate and adenosine 3,5-bisphosphate
FOXM1	Forkhead Box M1, critical proliferation-associated transcription factor
NDRG1	N-Myc Downstream Regulated 1, Stress-responsive protein involved in hormone responses, cell growth, and differentiation. Acts as a tumor suppressor
PLA2G4A	Phospholipase A2 Group IVA, catalyzes the hydrolysis of membrane phospholipids
BEX4	Brain Expressed X-Linked 4, functions as tumor suppressor
KIT	c-Kit/CD117, tyrosine kinase, plays a major role in melano-cytic migration
PLAU	Plasminogen Activator, Urokinase, associated with cell migration and invasion

Regulation of Cell Adhesion

Gene	Name and Function
GPR4	G Protein-Coupled Receptor 4, enables G protein-coupled receptor activity
PTK2B	cytoplasmic protein tyrosine kinase 2B, involved in calcium-induced regulation of ion channels
CYTIP	Cytohesin 1 Interacting Protein, sequesters Cytohesin-1 in the cytoplasm and thereby limits its interaction with β2 integrins, thereby reducing cell adhesion
LAMA5	Laminin subunit alpha-5, mediates the attachment, migration, and organization of cells by interacting with extracellular matrix proteins
PLAU	Plasminogen Activator, Urokinase, associated with cell migration and invasion
LAMA1	Laminin Subunit Alpha 1, extracellular matrix glycoprotein

RECK	Reversion Inducing Cysteine Rich Protein With Kazal Motifs, glycosylphosphatidylinositol-anchored glycoprotein that inhibits the enzymatic activities of matrix metalloproteinases	VTN	Vitronectin, cell adhesion and spreading factor
MMP-16	Matrix-Metalloproteinase 16, involved in the degradation of the extracellular matrix	TNXB	Tenascin-XB, glycoprotein of the extracellular matrix
ADAMTS16	ADAM Metallopeptidase With Thrombospondin Type 1 Motif 16, member of a family of multi-domain, zinc-binding proteinases		
MMP-1	Matris-Metalloproteinase 1, involved in the degradation of the extracellular matrix		
COL3A1	Collagen Type III Alpha 1 Chain, compound of the extracellular matrix		
COL5A3	Collagen Type V Alpha 3 Chain, fibrillar collagen		
