# Supplementary material for: Development of an optimized, non‐stem cell line for intranasal delivery of therapeutic cargo to the central nervous system
Source: Mol Oncol. 2023 Dec 26;18(3):528–46. doi: 10.1002/1878-0261.13569 (PMC10920084; doi:10.1002/1878-0261.13569)
Supplement: Supplementary file 1 — Fig. S1. Cell line authentication data. Fig. S2. Cell motility of PAR and FR cells determined by live cell imaging. Fig. S3. Proliferation of PAR, FR, and FR/TK cells. Fig. S4. GCV vulnerability of FR and FR/TK cells. Fig. S5. Uncropped immunoblots as shown in partial in Fig. 5. Fig. S6. Representative microphotographs of migrated LX2 cells from olfactory epithelium (OE) to the olfactory bulb (OB) of the mice. Fig. S7. In vivo in brain migration of shuttle cells. Table S1. Abbreviations, names and function of genes presented in Fig. 7C. [file MOL2-18-528-s001.zip › mol213569-sup-0009-Supinfo.pdf]

Supporting Information

Supplementary Figure 1: Cell line authentication data.

Supplementary Figure 2: A. Cell motility of PAR and FR cells determined by live cell imaging. Cell motility of 9-12 single cells per group was tracked over 24 h Accumulated (left graph), Euclidean (middle graph) distances as well as cell velocity (right graph) were calculated (n=3, SEM, t-test, n.s.: not significant, ** p<0.01). B. Microphotographs of the lower membrane side of a matrigel invasion chamber 6 h after seeding (scale bars: 100 µM). PAR: parental shuttle cells; FR: fast running shuttle cells; FR/TK: fast running, HSV-TK expressing shuttle cells.

Supplementary Figure 3: Proliferation of PAR, FR and FR/TK cells. 6.000 cells were seeded in microtiter plates and allowed to attach. 4 h after seeding and subsequently every 24 h later cell density was determined by staining the cells with crystal violet (n=3, SEM, t-test, * p<0.05; *** p<0.001). PAR: parental shuttle cells; FR: fast running shuttle cells; FR/TK: fast running, HSV-TK expressing shuttle cells).

Supplementary Figure 4: GCV vulnerability of FR and FR/TK cells. The cells were seeded in triplicates in microtiter plates and were treated with increasing concentrations of GCV for 72h. Cell density was determined by crystal violet staining (n=3; SEM; 2-way ANOVA, * p<0.05, ** p<0.01, *** p<0.001, **** p<0.0001). FR: fast running shuttle cells; FR/TK: fast running, HSV-TK expressing shuttle cells.

Supplementary Figure 5: Uncropped immunoblots as shown in partial in Figure 5.

Supplementary Figure 6: Representative microphotographs of migrated LX2 cells from olfactory epithelium (OE) to the olfactory bulb (OB) of the mice 6 h after INA (scale bar: 500 µM). PAR: parental shuttle cells; FR: fast running shuttle cells; FR/TK: fast running, HSV-TK expressing shuttle cells; INA: intranasal application.

Supplementary Figure 7: In vivo in brain migration of shuttle cells***.*** NSG mice were treated as indicated in Fig. 3. The enrichment of PAR, FR and FR/TK cells over time in different brain areas is presented in this figure (n=3-4 mice per group, 6-8 slices per mouse and brain area were quantified). PAR: parental shuttle cells; FR: fast running shuttle cells; FR/TK: fast running, HSV-TK expressing shuttle cells.

Supplementary Table: Abbreviations, names and function of genes presented in Figure 7C.
